# Association tests for rare and common variants based on genotypic and phenotypic measures of similarity between individuals

**DOI:** 10.1186/1753-6561-5-S9-S89

**Published:** 2011-11-29

**Authors:** Anbupalam Thalamuthu, Jingyuan Zhao, Garrett Teoh Hor Keong, Venkateswarlu Kondragunta, Indranil Mukhopadhyay

**Affiliations:** 1 Human Genetics, 60 Biopolis Street 02-01, Genome Institute of Singapore, Singapore 138672; 2 Internal Medicine, Eli-Lilly and Company, Lilly Corporate Center, Indianapolis, IN 46285, USA; 3 Human Genetics Unit, Indian Statistical Institute, 203 Barrackpore Trunk Road, Kolkata 700108, India

## Abstract

Genome-wide association studies have helped us identify thousands of common variants associated with several widespread complex diseases. However, for most traits, these variants account for only a small fraction of phenotypic variance or heritability. Next-generation sequencing technologies are being used to identify additional rare variants hypothesized to have higher effect sizes than the already identified common variants, and to contribute significantly to the fraction of heritability that is still unexplained. Several pooling strategies have been proposed to test the joint association of multiple rare variants, because testing them individually may not be optimal. Within a gene or genomic region, if there are both rare and common variants, testing their joint association may be desirable to determine their synergistic effects. We propose new methods to test the joint association of several rare and common variants with binary and quantitative traits. Our association test for quantitative traits is based on genotypic and phenotypic measures of similarity between pairs of individuals. For the binary trait or case-control samples, we recently proposed an association test based on the genotypic similarity between individuals. Here, we develop a modified version of this test for rare variants. Our tests can be used for samples taken from multiple subpopulations. The power of our test statistics for case-control samples and quantitative traits was evaluated using the GAW17 simulated data sets. Type I error rates for the proposed tests are well controlled. Our tests are able to identify some of the important causal genes in the GAW17 simulated data sets.

## Background

Genome-wide association studies have helped us to understand the genetic basis of several complex diseases and have identified thousands of variants associated with such diseases [http://www.genome.gov/gwastudies]. However, these variants explain only a small proportion of the phenotypic variance or heritability of a trait [1,2]. The fraction of heritability left unexplained might be determined by more common variants with small effect sizes, yet-to-be-identified rare variants with moderate to high effect sizes, and other types of variants, such as copy number and complicated structural variants. Next-generation sequencing technologies might help us to identify rare and functionally relevant variants through targeted resequencing and whole-genome sequencing. Through resequencing, rare variants can be shown to be associated with several phenotypes [3,4]. Currently, a number of exome sequencing studies aimed at identifying rare functional variants are under way. Because the cost of sequencing is falling rapidly, in the near future we can expect investigators to undertake whole-genome sequencing studies to characterize all the variants that determine many phenotypes.

One active area of research in statistical epidemiology is the development of efficient statistical tests to detect associations involving rare variants. In resequencing studies, the number of variants available for testing within a genomic region is generally large. Selecting rare variants for association tests presents a challenge. Using statistical tests of association for each rare variant is not an optimal strategy because of multiple test issues and the typically low statistical power of the test for rare variants. Therefore many of the procedures proposed in the literature involve some kind of pooling or the use of a weighted combination of the rare variants to establish the joint association [5]. King et al. [6] incorporated the fitness effects of rare variants into association testing, under the framework of mixed effects linear models. Bhatia et al. [7] proposed a method of association testing for rare variants, referred to as the covering method, that is based on a subset of variants that achieve maximum discrimination between case subjects and control subjects. Zawistowski et al. [8] proposed a simple pooling technique based on cumulative minor allele counts, which can also be used for imputed rare variants (e.g., rare variants imputed based on the 1000 Genomes Project).

Recently, we developed a test statistic, the kernel-based association test (KBAT), to test the joint effect of multiple variants based on the genetic similarity between individuals [9]. In the current study, we modify this method to include rare variants and to extend it for multiple subpopulations. Our test statistic can be based on either only rare and functionally relevant variants or both rare and common variants to determine their synergistic effects [3]. Also, to test the association of quantitative traits, we propose a new test statistic, which we refer to as the quantitative trait KBAT (QT-KBAT); this statistic is based on genotypic and phenotypic measures of similarity between pairs of individuals. We then use the KBAT and the newly proposed QT-KBAT statistic to test the association of rare and common variants with both binary and quantitative traits. To evaluate the performance of the two test statistics, we use the Genetic Analysis Workshop 17 (GAW17) simulated data sets.

## Methods

Here, we present methods for testing the joint association of several rare and common variants with binary and quantitative phenotypes. To construct the test with all variants, rare variants are pooled and common variants are considered individually. We introduce first a pooling strategy used for rare variants, followed by the measures of genotypic and phenotypic similarity between individuals. Next we describe the KBAT statistic, which is based on pooled rare variants and common variants for multiple subpopulations. Finally, we present the statistic for quantitative traits (the QT-KBAT).

### Pooling of rare variants

Suppose that genotyping is done for *n* individuals on *K* single-nucleotide polymorphisms (SNPs) within a genomic region or gene. To test the joint association of all SNPs, the rare SNPs are pooled, as described in what follows.

Let  denote the genotype code (the number of minor alleles) at the *k*th SNP for the *i*th individual. Based on a minor allele frequency (MAF) threshold, without loss of generality we assume that 1 to *K_r_* are rare SNPs and that the rest, *K_c_* = *K* − *K_r_*, are common SNPs. The combined genotype of the rare variant is given by:(1)

The pooled genotype is the total number of minor alleles among the rare variants truncated at 2. For the gene-level test, the truncation does not involve much loss of information because the proportion of individuals with more than two rare variants within a gene is usually small. This coding is only a convenience and not a constraint for the current analysis. Other types of pooling strategies can be devised based on the nature and function of the SNPs. However, in tests with several rare variants, as in a pathway-level or whole-genome-level test, different thresholds for pooling can be used. But, this issue is not discussed here because it is beyond the scope of this paper.

### Similarity measures

We use two types of similarity measures: the allele match kernel for genotype similarity and the Euclidean distance for phenotype similarity.

For any SNP (pooled rare or common), the allele match kernel score determines the number of alleles common to genotypes *g_i_* and *g_j_* of two individuals *i* and *j*. The score is 4 if *g_i_* and *g_j_* are the same, 2 if one is a heterozygote and the other a homozygote, and 0 if they do not share any common alleles (Table 1). The power of the allele match kernel score is comparable to several other kernel similarity functions. This kernel is similar to the identity-by-state allele-sharing kernel function used by other researchers (see Mukhopadhyay et al. [9] for details).

**Table 1:** Similarity score between a pair of genotypes *g_i_* and *g_j_* using the allele match kernel

	Allele match for *g_i_*		
	
*g*_*j*_	0	1	2
0	4	2	0
1	2	4	2
2	0	2	4

Let *T_i_* = (*t*_1_*_i_*, *t*_2_*_i_*, …, *t_Qi_*) and *T_j_* = (*t*_1_*_j_*, *t*_2_*_j_*, …, *t_Qj_*) denote a vector of *Q* quantitative trait values for two individuals *i* and *j*. The square of the Euclidean distance between two vectors is used to define the phenotypic similarity between two individuals, which is given by:(2)

Two individuals who have similar trait values will therefore have a lower similarity score compared to individuals with different trait values. Although there are several measures of phenotypic similarity, only the Euclidean distance metric is considered here.

### Test statistic for case-control samples (KBAT)

Assume that we have genotypes for *n*_1_ case subjects and *n*_2_ control subjects for *K* SNPs. Details of the KBAT statistics for testing the joint association of all *K* SNPs with the disease are given by Mukhopadhyay et al. [9]. Here, we briefly present the test statistic based on pooled rare variants and common variants.

For the *k*th SNP, , where *P* denotes the subscripts for the pooled rare variants , which is the genotypic similarity between two individuals *i* and *j* within group *l* (*l* = 1 denotes a case subject and *l* = 2 denotes a control subject) with the respective genotypes  and , respectively. We use the *U* statistic, denoted as:(3)

The within-group sum of squares (SSW) is represented by:(4)

The between-group sum of squares (SSB) is represented by:(5)

where:(6)

and:(7)

The statistic:(8)

based on the analysis of variance (ANOVA) model, is used to test the joint effect of all SNPs with the disease status.

### Test statistic for quantitative traits (QT-KBAT)

Based on the measures of genotypic and phenotypic similarity, we introduce a KBAT-type method to test the association of genotypes with quantitative phenotypes. For a SNP *k*, consider the genotypic similarity scores of the allele match kernel, given in Table 1. There are only three possible similarity values between any two individuals; hence all possible pairs of individuals from *n* samples can be assigned to one of these three similarity groups. Individuals from group 1 are those pairs whose genotype similarity value is 4, that is, those pairs with genotypes (0, 0), (1, 1), and (2, 2). Individuals from groups 2 and 3 can be similarly identified.

Let:(9)(10)

and:(11)

denote the pairs of individuals in groups 1, 2, and 3, respectively. Then, the number of individuals in these groups is given by:(12)(13)

and:(14)

respectively, where *f*_0_, *f*_1_, and *f*_2_ denote the frequency of the genotype counts 0, 1, and 2, respectively, for SNP *k*. Note that the number of groups *m* (here *m* = 3) and the genotype pairs within the groups can vary depending on the definition of the kernel function used to score the genotype similarity.

If genotypes are associated with the trait values, then a strong correlation would be expected between the genotypic and the phenotypic similarity. Therefore the average similarity for the pairs across these three groups should be different. In general, a higher average phenotypic similarity is expected in groups with higher genotypic similarity. Thus genotypic similarity is compared across three groups using the one-way ANOVA model, as in KBAT. Consider the model:(15)

Here,  denotes the phenotypic similarity for the pair (*i*, *j*) in group  based on the similarity group induced by the *k*; *μ* is the overall mean or the general effect for pairs of individuals; *α_l_* denotes the group-specific treatment effect for similarity scores over the general effect; and *e_l_*_(_*_ij_*_)_ are the error components.

To test the joint association of the *K* SNPs with the traits, the QT-KBAT statistic is defined as:(16)

where:(17)

and:(18)

denote the within-group sum of squares and the between-group sum of squares, respectively, for SNP *k*,(19)(20)

and(21)

The usual assumptions of the ANOVA model are not valid because the observations within each of the groups are correlated. In addition, the distribution of phenotypic similarity values, as defined earlier (Eq. 2), may not follow a normal distribution. Therefore permutations are used to compute the *p*-value of the ℑ statistic in Eq. (16). The test statistics for the quantitative trait are slightly different from the KBAT statistics for case-control samples (sum of *F* ratios compared to the ratio of the sum of the SSBs to the sum of the SSWs). Although it is straightforward to define QT-KBAT similarly to KBAT, the form in Eq. (16) is used so that the *F* statistics of the one-way ANOVA from any standard computer program can be directly used.

### Extension to multiple subpopulations

Suppose that samples of sizes *N_i_* (*i* = 1, 2, …, *L*) are obtained from *L* different populations. When performing tests of association using regression models, it is possible to use subpopulation indicator variables to account for population stratifications. Here, we adopt a similar approach by taking the weighted combination of the population-specific *F* statistics. The combined statistic for the whole data set is given by:(22)

where:(23)

*i* = 1, 2, …, *L*, and the ℑ_*i*_ are the population-specific case-control (KBAT) or quantitative trait (QT-KBAT) association statistics defined by Eq. (8) or Eq. (16), respectively.

## Results

The GAW17 mini-exome genotype data consist of subsamples from several ethnic cohorts. The samples from Europeans, Asians, and Africans are well separated in the plot of principal components derived with and without rare variants (data not shown). From the simulation model, we know that the phenotypes were simulated without reference to the ethnicity of the samples. However, we performed the analysis twice, once assuming a single population and once assuming three subpopulations (Europeans, Asians, and Africans). Furthermore, we considered two categories of SNPs: (1) only nonsynonymous SNPs, because the phenotype simulations are based on only nonsynonymous SNPs; and (2) all SNPs, to examine changes in the power of the test statistic for additional noncausal variants. We used the KBAT statistic to test the association of genes with case-control status, and we used the QT-KBAT statistic to perform three separate tests for the quantitative traits, namely, Q1, Q2, and a multivariate trait (Q1, Q2, Q4). There are 3,205 genes for the all-SNPs analysis and 2,194 genes for the nonsynonymous-SNPs-only analysis. For the case-control analysis (KBAT) we tested all the genes, and for the quantitative traits analysis (QT-KBAT) we tested only the causal genes (9 for Q1, 13 for Q2, and 36 for the multivariate trait analysis; see Almasy et al. [10] for the details of the simulation model). As noted, we performed the analysis twice. Here, we discuss only the results corresponding to the analysis assuming a single population. The complete set of results can be obtained from the corresponding author.

### Pooling

For a given set of SNPs within a gene, we first pooled all rare SNPs (MAF < 0.05) and no pooling was done for common SNPs. For example, if a gene contained seven rare SNPs and three common SNPs, then our gene-level test would have four SNPs (one pooled using the seven rare SNPs and the three common SNPs). We used a pooling MAF threshold of 0.05 because we wanted to have a reasonable frequency of the three counts of the kernel score for pooled SNPs for our QT-KBAT statistics. A reduced threshold increases the number of SNPs for our test statistics and hence may reduce the power.

### Type I error

We also checked the type I error rates of the KBAT and QT-KBAT statistics. For type I error computation, we first selected one of the 200 replicates of phenotypes at random and then permuted the original phenotype; *p*-values for the permuted data set were obtained with an additional 2,000 permutations of the phenotypes.

### Power

For each of the 200 iterations, we computed the *p*-values of association tests using 2,000 permutations of the trait values. Assuming that the phenotypes in the 200 iterations were generated under the alternative hypothesis, the power of the test at a 5% level of significance was computed here as the proportion of times the unadjusted *p*-values were less than 0.05 out of the 200 iterations.

### Case-control association

**Figure 1:**
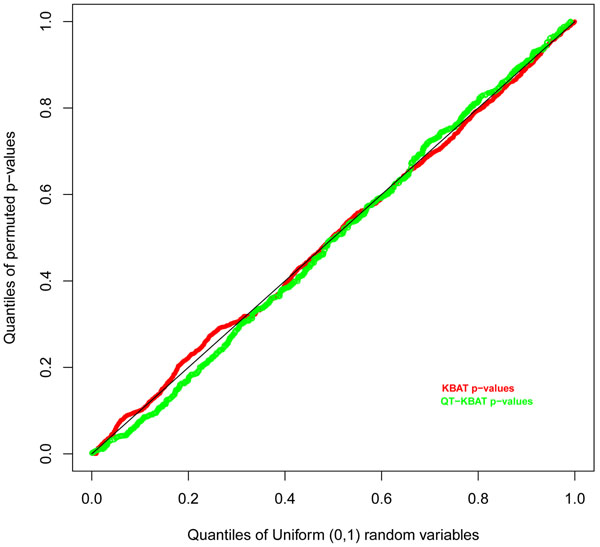
**Quantile-quantile plot of *p*-values of KBAT and QT-KBAT based on permutations and the expected *p*-values assuming uniform distribution.** Restricting the analysis to nonsynonymous SNPs, we calculate the *p*-values for the case-control and quantitative trait associations based on 2,000 permutations. The plot for quantitative traits is based on the association test with the trait Q1 for a sample of 1,000 genes, and for the case-control test the plot is for all 2,194 genes with at least one nonsynonymous SNP. For each gene the *p*-value is calculated using a random selection of one of the 200 replicate data sets.

**Figure 2:**
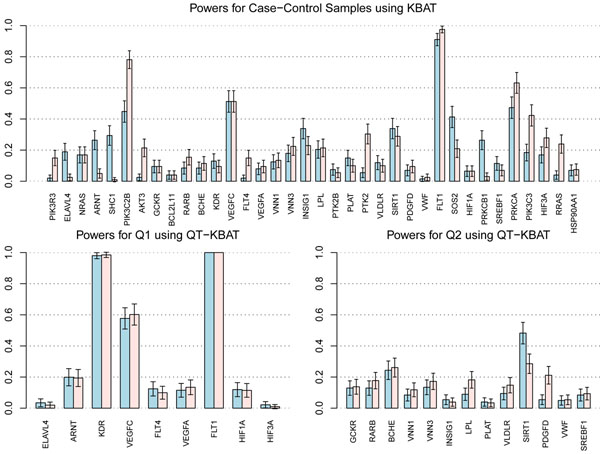
**Powers of KBAT and QT-KBAT at the 5% level of significance**. Powers (*y*-axes) for case-control (KBAT) and quantitative traits (QT-KBAT) association tests with simulated causal genes are plotted. Analysis including all SNPs (light blue) and analysis of only nonsynonymous SNPs (rose) are plotted side by side with error bars based on 200 replications.

Type I error rates for the KBAT statistic for rare variants were well controlled (Figure 1). To understand the false-positive rate for the KBAT statistic, we first removed the 695 spuriously associated genes reported by Luedtke et al. [11]. Therefore we were left with 1,499 and 2,510 genes, respectively, for the analyses of nonsynonymous SNPs and all SNPs. With a set power threshold of 0.4 to declare significance, in the nonsynonymous SNPs analysis we found 68 associated genes above this threshold, of which 5 were causal under the simulation model. Therefore we had 63 false positives out of 1,499 genes, which represents approximately 4%. In the analysis considering all SNPs, we found 5 causal and 131 false-positive genes with power greater than 0.4, which gave a false-positive rate of approximately 5%. The power of the KBAT statistic for all 36 causal genes is shown in Figure 2.

### Quantitative traits association

The quantile-quantile (Q-Q) plot based on the random sample of 1,000 genes shows that the type I error rate for QT-KBAT is well under control (Figure 1). We restricted the analysis to only the causal genes to examine the power of the QT-KBAT statistic. The powers for the quantitative traits Q1 and Q2 with the causal genes is plotted in Figure 2. There are three genes with power greater than 0.4 for Q1 for both the nonsynonymous-SNPs-only and all-SNPs analyses, but only one gene for Q2 under the all-SNPs analysis. For the multivariate trait, we tested all 36 causal genes and found powers of 0.984 for gene *FLT1*, 0.751 for *KDR*, 0.338 for *VEGFC*, and 0.29 for *PIK3C3*.

The Q-Q plot shown in Figure 2 is only an approximation because the distribution of the test statistic for different genes is not the same as a result of variation in the number and frequency spectrum of the variants within the genes. Furthermore, we could not examine the false-positive associations for the quantitative trait because not all the genes were tested. However, among the case and control subjects there were several noncausal rare variants with genotype frequency similar to the causal variants. These noncausal variants automatically generated association with the phenotypes, which were not false-positive but latent associations. Hence the false-positive calculations using this data set may not be accurate.

## Discussion

We proposed joint association tests for both rare and common variants in a genomic region. A comparison of the analysis of all SNPs with the analysis involving only nonsynonymous SNPs showed that the inclusion of additional nonassociated SNPs in general reduced the power of the test. However, if moderately common variants are close to the rare causal variants, it is possible that the latent correlation between these common and rare variants may improve the power of the test when these common variants are included. For example, the gene *SIRT1* has improved power for all SNPs because this gene has a synonymous SNP (C10S3059) with MAF = 0.167, which has an *r*^2^ value of 0.0142 (maximum *r*^2^ value for this SNP with all others within this gene) with the causal variant C10S3110 with MAF = 0.002. Similarly, the higher power values observed with the inclusion of all SNPs in genes *ARNT*, *SHC1*, and *INSIG1* may be due to these genes having synonymous common variants with MAFs of 0.43, 0.09, and 0.04, respectively. Therefore in some cases the synergic effect of common and rare variants may have improved the power, and we thus suggest examining the powers of the two analyses: one that considers only the rare variants and the other that includes both rare and common variants.

Our quantitative association test can be used for testing the pleiotropic effect of genes on multiple phenotypes. Our multivariate analysis did not identify any additional causal genes because the GAW17 simulation model treats each trait independently with separate sets of causal genes. In future studies we will examine the power of this approach for testing pleiotropy.

Because of time constraints, our analysis was restricted to only the allele match kernel for scoring the genotypic similarity and the squared Euclidean distance for scoring the phenotypic similarity between individuals. However, several choices of kernel functions for determining genotypic and phenotypic similarity could be examined. It is important to identify optimal similarity metrics, because the interpretation of the analysis and its power may depend on the similarity measure used.

The association tests proposed here are not adjusted for other covariates. However, we could perform a stratified analysis on the subcategories of certain covariates if sufficient samples were available. We are also exploring the possibility of testing the joint association of both quantitative and binary traits using a single test statistic. In addition, we have not included any weights, such as the frequency or functional weights, of rare variants. Furthermore, we used a MAF threshold of 0.05 for pooling rare variants. In our method, implementing various subsets for pooling SNPs, such as using MAF thresholds and the nature of SNPs, is straightforward.

The permutation procedure for the case-control test statistic KBAT can be efficiently implemented. Using the KBAT method, we have performed a gene-based analysis even for data sets from genome-wide association studies. The current implementation of the QT-KBAT to compute the *p*-value using permutation takes a long time if there are many SNPs. Therefore we restricted the quantitative trait analysis to only 36 causal genes. We are currently exploring the possibility of efficiently implementing the permutation procedure for QT-KBAT in a genome-wide analysis.

One of the main drawbacks of our QT-KBAT statistics is the unequal distribution of observations within groups. The number of observations in each group depends on the allele frequency of the SNPs (either pooled or common), and if the MAF is small, then one of the group sizes may become small or zero.

## Conclusions

Future genetic association studies for common complex genetic disorders will involve the analysis of rare and common variants. Therefore efficient statistical techniques are needed for their integrated analysis. In the current study we proposed methods for testing the joint association of rare and common variants that underlie quantitative and qualitative phenotypes. We developed a test statistic using measures of genotypic and phenotypic similarity between two individuals under the ANOVA framework. This approach can also be used for combining multiple phenotypes and multiple subpopulations. We applied our statistics to the GAW17 simulated data sets and identified important simulated causal genes.

## Competing interests

The authors declare that there are no competing interests.

## Authors’ contributions

AT conceived of the study, performed the statistical analysis and wrote the manuscript. JZ, GTHK participated in the design and statistical analysis. VK, IM participated design of the study, and in the draft of the manuscript. All the authors read and approved the final manuscript.
